# Service Migration from Cloud to Multi-tier Fog Nodes for Multimedia Dissemination with QoE Support

**DOI:** 10.3390/s18020329

**Published:** 2018-01-24

**Authors:** Denis Rosário, Matias Schimuneck, João Camargo, Jéferson Nobre, Cristiano Both, Juergen Rochol, Mario Gerla

**Affiliations:** 1 Computer Science Faculty, Federal University of Pará, Belém 66075-110, Brazil; 2 Institute of Informatics, Federal University of Rio Grande do Sul, Porto Alegre 91501-970, Brazil; makschimuneck@inf.ufrgs.br (M.S.); jvcamargo@inf.ufrgs.br (J.C.); juergen@inf.ufrgs.br (J.R.); 3 Polytechnic School, University of Vale do Rio dos Sinos, São Leopoldo 93022-750, Brazil; jcnobre@unisinos.br; 4 Federal University of Health Sciences of Porto Alegre, Porto Alegre 90050-170, Brazil; cbboth@ufcspa.edu.br; 5 Computer Science Department, University of California Los Angeles, Los Angeles, CA 90095-1596, USA; gerla@cs.ucla.edu

**Keywords:** fog computing, QoE, service migration

## Abstract

A wide range of multimedia services is expected to be offered for mobile users via various wireless access networks. Even the integration of Cloud Computing in such networks does not support an adequate Quality of Experience (QoE) in areas with high demands for multimedia contents. Fog computing has been conceptualized to facilitate the deployment of new services that cloud computing cannot provide, particularly those demanding QoE guarantees. These services are provided using fog nodes located at the network edge, which is capable of virtualizing their functions/applications. Service migration from the cloud to fog nodes can be actuated by request patterns and the timing issues. To the best of our knowledge, existing works on fog computing focus on architecture and fog node deployment issues. In this article, we describe the operational impacts and benefits associated with service migration from the cloud to multi-tier fog computing for video distribution with QoE support. Besides that, we perform the evaluation of such service migration of video services. Finally, we present potential research challenges and trends.

## 1. Introduction

A wide range of multimedia services is expected to be offered for mobile users via various wireless access networks [1]. Forecasts for 2020 indicate that multimedia transmission will represent up to 90% of the global IP data traffic [2], where video delivery over mobile wireless networks will take about 50% of the overall global data traffic [3]. To cope with growth of multimedia traffic of wireless networks, the next generation of mobile and wireless networking technologies, such as, 5 G wireless networks, mobile-edge computing (MEC), software-defined networking (SDN), and cloud radio access networks (Cloud RAN) are been designed to attain a 1000-fold capacity increase, 5 times reduced latency, and 10 times longer battery lifetime.

In this way, the integration of cloud computing in such wireless multimedia environments provides a set of optimization services that demand high computing and energy resources, such as video quality assessment, cache, load balancing, Adaptive Bit Rate (ABR), and others [4]. These services might operate in a cloud computing environment, which consists of data center infrastructure with high computing resources accessible across the Internet. However, in airports, railway stations, vehicular networking infrastructures, and sports stadiums, thousands of users uploading/downloading multimedia content with high traffic and similar user demands and location from the cloud quickly outstrip the bandwidth capacity, increase the delay, and deliver videos with poor Quality of Experience (QoE) [5]. In such scenario, the network conditions impact Video on Demand (VoD) services regarding initial buffering time, re-buffering events, re-buffering duration, playback start time, the average playback bitrate, and also the variability of the bitrate [6,7].

Instead of concentrating data and computation in a small number of large clouds, fog computing considers that portions of the cloud services must migrate to fog nodes located at the network edge, meeting the user needs regarding delay and QoE. The main characteristic of fog computing is its topology, i.e., the geographically distributed nodes that perform computation and offer storage and network services [8]. The basic elements of fog computing, called fog nodes, can be designed and deployed in many ways to meet the user needs best, where fog nodes can be located in a logical and physical hierarchy, arranged in layers between the cloud on top and the mobile devices at the bottom [9]. Specifically, fog nodes might range from dedicated servers in the core network to the mobile devices, i.e., such fog nodes have different characteristics regarding processing and storage, and their capacities are growing exponentially by following the Moore Law [10]. In addition, fog resources exist in different domains, including communication, computation, and storage, which can be exploited as needed by users. The hierarchical organization of the fog allows the processing, networking, and storage to be carried out at each level to match the topology and distributed workload properties of the applications running on the fog node [9].

Fog computing has been conceptualized to facilitate the deployment of new services that cloud computing cannot provide, particularly those demanding QoE guarantees [11]. Fog nodes are capable of virtualizing their functions/applications, and multiple applications can run concurrently [12]. In this way, fog nodes might offer video optimization services, such as ABR and caching schemes, to improve the QoE of transmitted videos. For instance, the QoE of delivered videos can be enhances with ABR streaming provided by fog nodes, where the video is adapted according to the different network, device, and user characteristics [6,13]. Furthermore, smart caching available on fog nodes reduce the traffic load and delay, since viral videos might be available closer to the users [14,15].

To the best of our knowledge, existing works on fog computing focus on architecture and fog node deployment issues [9,10,16,17]. Specifically, fog node deployment must ensure that mobile users have access to fog services with low delay, QoE support, and without significant network overhead [18]. However, portions of the cloud services must migrate to fog nodes located at the network edge, meeting the user needs regarding delay and QoE, which is still an open issue due to user mobility, request patterns, and the time needed to migrate the service [19,20]. Hence, one of the key design issues in fog computing is the service migration from the cloud to multi-tier fog nodes in the case of poor QoE. The service migration must achieve the trade-offs among cost, network overhead, and QoE to bring benefits for both user and network/content provider. It is important to mention that specific algorithms for service migration must consider the trade-offs between the available resources in each tier and the performance gains provided by each tier since fog nodes have different computation availability [21].

In this article, we describe the operational impacts and benefits associated with service migration from the cloud computing to multi-tier fog nodes for video distribution with QoE support. In this way, we introduce a multi-tier fog computing architecture based on SDN paradigm for video distribution. Network devices have different characteristics about processing and storage, where these devices can act as fog node to provide video optimization services, such as ABR and caching schemes, closer to the user, meeting its requirements as delay and QoE. The proposed architecture provides the cooperation between fog and cloud to run video services any VoD service provider, giving satisfactory QoE.

In the proposed architecture, the cloud distributes video content and also orchestrates/controls fog nodes in a centralized fashion, while multi-tier fog nodes run multimedia services to meet user requirements. It is important to mention that besides we focus on video distribution, the proposed architecture can be adapted for other services, e.g., traffic monitoring and navigation for an autonomous vehicle, wearable applications, augmented reality, and others, that requires fog computing and service migration. The main contributions of this article are: (i) a multi-tier fog computing architecture to enable video distribution with QoE Support; (ii) a fog-enabled service migration in such architecture; (iii) the evaluation of a service migration to minimize the traffic in the core network; and (iv) the identification of potential research challenges, opportunities, and requirements for such fog environments. Based on the performed experiments, the service migration improves the QoE compared to the traditional cloud architectures for multimedia distribution.

The remainder of this article is organized as follows. In Section 2, we introduce a fog computing scenario. In Section 3, we present the proposed QoE-aware service migration scheme and the multi-tier fog architecture. In Section 4, we introduce an experimental case study to analyze the performance of the service migration in the proposed architecture. In Section 5, we present the key research challenges related to multimedia distribution combined with fog computing. Finally, the concluding remarks are presented in Section 6.

## 2. Shifting the Multimedia Load from the Cloud Computing to the Fog Nodes

Human behavior is changing from traditional communication paradigm based on voice calls or text messages to real-time video services transmitted over mobile devices, such as Smartphone, Tablets, and Notebooks [22]. In this way, emerging multimedia applications, e.g., Video on demand, interactive 3D, high-definition, or even 4k/8k ultra-high-definition (UHD) video streaming, will flood the next generation of mobile and wireless networking technologies, requiring unprecedented high access speed and low latency [23]. In such applications, users expect to watch videos anytime and anywhere with QoE support, where QoE be can defined as the overall acceptability of an application or service for the end-user [24]. Therefore, the QoE of delivered videos can be improved with ABR and cache schemes running on fog nodes.

ABR streaming delivers adapted videos according to the different network, device, QoE, and user characteristics. Several ABR schemes have been developed, such as Apple HTTP Live Streaming (HLS), and Dynamic Adaptive Streaming over HTTP (DASH), among others [25]. In ABR streaming, each video is divided into multiple chunks, and each chunk can be requested with a different bitrate version of the video to avoid buffer underflow, preventing stalling in varying wireless network conditions. The video client might request an appropriate bitrate version based on current network conditions, where it selects lower bitrate at the video beginning. The bitrate increases as soon as the network conditions become better, and reduces in case of buffer underflow [26].

In hot spots (e.g., airports), a group of users located in the same area, sharing the same preferences or wireless network might search and watch hit videos, causing duplicated downloads [27]. For instance, studies [28,29] showed that parts of traffic load are due to the download of some popular contents, e.g., 10% of the top favorite videos account for nearly 80% of views. In this way, cache schemes available on fog nodes reduce redundant content transmissions, enabling users to access popular content from caches located closer to the user, decreasing the need of user access to the service provider [14].

It is now widely agreed that relying only on cloud computing to provide UHD video streaming is inadequate to provide QoE support [10]. Furthermore, the data exchange between users and clouds will allow the “data tsunami", which saturate and bring down the backhaul networks, as well as increase the delay. In this way, it is essential to supplement cloud computing with fog computing to pushes traffic, computing and network function towards the network edges [30]. Fog nodes are capable of virtualizing their functions, where multiple applications can run concurrently [9]. Virtualization technology enables a single fog node to provide computing services to multiple mobile devices, by creating multiple instances of the virtualized service for simultaneously performing different tasks or operating different applications. In this way, portions of the cloud services could migrate to fog nodes located at the network edge, meeting the user needs about delay and QoE.

## 3. The Migration of Video Services from the Cloud Computing to Multi-Tier Fog Nodes

In this section, we introduce a multi-tier fog computing architecture based on the SDN paradigm to distribute video with QoE support. The cloud infrastructure and fog nodes work collaboratively, in which the cloud migrate multimedia services to multi-tier fog nodes in case of poor QoE. In this context, video service migration requires modification either in the computing resources (e.g., the provisioning of a virtualized service on a given tier) or the networking infrastructure (e.g., installing new flow rules after a service migration).

### 3.1. Multi-Tier Fog Architecture to Provide Service Migration

**Figure 1:**
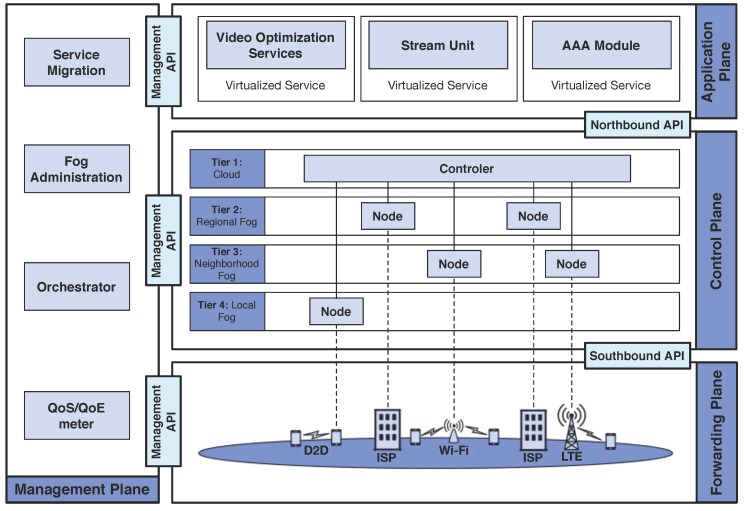
Multi-tier Fog Architecture.

Figure 1 introduces an architecture composed of a cloud computing (Tier 1) together with multi-tier fog nodes (Tiers 2, 3, and 4), which work collaboratively to enable service migration for video distribution with QoE support. In such architecture, we consider fully connected and fully fog-enabled scenario, where fog nodes are hierarchically organized to provide video services for end-users. There may be widely distributed local fog nodes, e.g., mobile devices (i.e., Tier 4), where such fog node relays the video content via device-to-device (D2D) wireless communication for mobile devices with high and similar traffic demands could cooperate with each other to form a D2D network. The neighborhood fog node, e.g., Base Station (BS) or Access Point (AP) (i.e., Tier 3), supports a few dozen to perhaps a few hundred local fog nodes. Above these would be regional fog node, e.g., baseband unit (BBU) or Internet Service Provider (ISP) (i.e., Tier 2), managing city-wide coordination. On the top of such multi-tier architecture, there is the cloud (i.e., Tier 1).

In such architecture, the cloud distributes video content and also orchestrates/controls the system behavior in a centralized fashion. On the other hand, fog nodes might be deployed in different levels at the network edge to provide video services closer to the users. The client requests the video content for the service provider at the cloud, and then displays for the user. Such architecture requires flexibility, programmability, and management for integrated network-centric operations, where SDN paradigm addresses such capabilities [31,32]. Hence, the proposed architecture follows the key ideas of the SDN paradigm [33], since it is convenient to implement the SDN on Cloud-based network application because of its natural character of a centralized control mechanism.

SDN is a promising paradigm, which provides flexibility to network management by separating the network infrastructure into distinct planes, where each plane can be programmed to meet particular application requirements [34]. Specifically, one of the main features of SDN is the separation of the control plane and data plane, and thus the network elements become simple forwarding devices and the control logic is implemented in a controller. This separation simplifies policy enforcement and network configuration, evolution, scalability, as well as the control and data planes can be developed separately from each other. SDN also considers a centralized entity (called SDN controller) that coordinates the forwarding decisions of network elements [35]. In this way, by considering a programmable SDN controller, the network operators can easily configure new network devices, quickly deploy new applications, and migrate services between network nodes [10]. SDN enables the programmability of the control plane, which refers to the ability of control, change, and manage network policies on-the-fly employing software via open interfaces in contrast to relying on closed boxes and proprietary defined interfaces.

By following the SDN paradigm, the proposed architecture consists of four planes (application, control, forwarding, and management planes), three APIs (northbound, southbound, and management interfaces), and a centralized controller. The southbound API allows the forwarding plane to communicate with the control plane, where OpenFlow [36] is the current well-known de facto standard protocol adopted by industry and used in the southbound interface. The northbound API abstracts control plane functions to network applications at the top level, where applications can exchange data with the SDN controller, e.g., applications use this interface to communicate their requirements to the SDN controller or to get information about the overall state of the network. The management API allows information to flow between the management plane and other planes [32]. The details of each layer are described as follows.

The application plane is on the top of the proposed architecture to support applications to perform different tasks. However, these applications have different requirements in terms of QoE, QoS, and security levels, which might be considered. For instance, this plane can be composed of the following applications: Stream Unit, Video Optimization Service, Authentication, Authorization, and Accounting (AAA). These modules/applications run on independent virtualized services, where the virtualization technology enables to migrate services between different tiers of the proposed architecture. The proposed architecture works with any virtualization technology, such as Virtual Machine (VM) or containers. Fog nodes do not have substantial resources as in the cloud, and thus the virtualized technology must be lightweight to provide quick deployment with low overhead, as well as enabling support for live service migration [12]. In this context, a single edge device (i.e., a fog node) can provide computing services to multiple mobile devices by creating multiple virtualization instances for simultaneously performing different tasks or operating different applications.

The stream unit application distributes video content for each client request, where the client downloads the video and shows the content to the user. This application must be compatible with existing video players on mobile devices, such as ffplay [37]. The application plane also supports different video optimization services, such as ABR and cache schemes, to improve the QoE of delivered videos. For instance, in the case of poor QoE, an ABR scheme at a given tier must adjust either the video codec, bit rate, or resolution according to the current network conditions and device capabilities [25]. Finally, the AAA module is responsible for fulfilling the security requirements. At the cloud side, it controls/tracks user and the consumed resources and services, and also interacts with other modules to perform security measures, such as auditing and billing. At the client side, it provides the authentication of mobile devices and users, the authorization for the consumption of networking and computational resources, and the accounting for the collection of relevant data. The control plane is located at the middle of the network architecture and it is the most important, since its functions determine the behavior and performance of the network. A centralized controller deployed in the cloud performs the control functions in a consolidated fashion as in a traditional SDN architecture. The controller enforces the adaptation/control actions taken by the orchestrator module, manages/provides communication among nodes, and synchronizes control/data flow exchange. As an example of the tasks performed through the control plane, the deployment of a fog node in a given tier to migrate ABR scheme from the cloud to a fog node can be managed by the controller. An event that can trigger such fog computing deployment is the detection of poor QoE.

The forwarding plane is located at the bottom of the SDN architecture and it is composed of (re)configurable nodes connected to a centralized controller. This plane is responsible for information maintenance, network information, and others. The cloud computing (Tier 1) distributes video content and also orchestrates/controls multi-tier fog nodes. A fog node might be deployed at the network edge in different levels: (i) ISP or BBU (Tier 2); (ii) BS or AP (Tier 3); and (iii) mobile devices (Tier 4). For instance, client might become a fog node to download and share the content among its neighbors using Wi-Fi direct, Bluetooth, or another D2D wireless technology.

Management plane supports the execution of administrative and operational tasks. Some examples of such tasks are the gathering of information from the forwarding plane as well as the generation of performance reports. The management plane is composed of the following modules: service migration, orchestrator, fog administrator, and QoE/QoS meter. The QoE/QoS meter enables collecting QoE and QoS measurements using well-established tools during video content displaying. For instance, CoLisEU tool [5] supports the gathering of QoS metrics (e.g., throughput, delay, Round Trip Time - RTT, and Received Signal Strength Indicator-RSSI) and QoE ones (e.g., playback start time, duration of freezes, and Mean Opinion Score-MOS). Besides QoS and QoE measurements, the QoE/QoS meter also provides feedback (device capability, content request, preferences, and channel condition) to help the orchestrator to take decisions according to user needs.

The orchestrator module is responsible for the decision-making that considers input from different sources, such as the QoS/QoE meter and operator-specific information (e.g., network policies and service level agreements). In this way, this module makes decisions about video adaptation, cache, service migration, and fog deployment. For instance, if the orchestrator aims to deploy a fog node, the service migration module is responsible for steering the corresponding virtualization service in a given tier. In this context, the fog administrator module manages the instantiated fog nodes and distributes multimedia content in different fog nodes.

### 3.2. Video Service Migration

Video services migration scheme can provide video dissemination with QoE support using multi-tier fog nodes. For instance, video service migration can be triggered by user mobility, energy conservation, traffic load reduction, or service replacement. In the proposed architecture, the cloud, i.e., Tier 1, stores, processes, and distributes adapted video content for each client request. The client sends feedback to the orchestrator at the cloud, which takes decisions according to user needs. These decisions aim at minimizing degradation of end-to-end streaming quality in the face of network and user dynamics.

**Figure 2:**
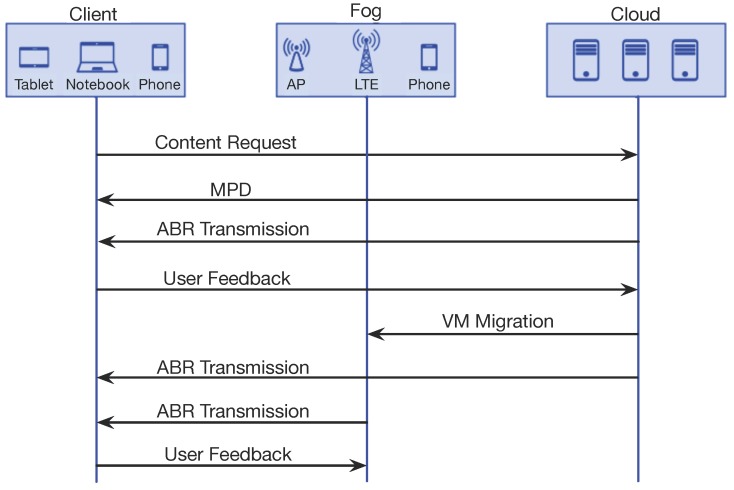
Video Service Migration to Multi-tier Fog Nodes.

Figure 2 shows a video services migration scheme. First, the client requests some content to the cloud. Next, the streaming unit sends the Media Presentation Description (MPD), which is an XML file that containing the information about video segments, as well as their relationships and information required for the client to choose between them. It also includes other metadata needed by clients to select the media segments from the streaming unit. Afterwards, the content is exchanged between the client and the cloud using ABR. In this context, user feedback sent to the cloud can trigger the service migration to a fog node, e.g., due to QoE issues. The transmission can be provided both by cloud and fog node. Finally, the client can send additional feedback to the fog node that may lead to another service migration, either to a (new) fog node in a different tier or even to the cloud.

Virtualization technology allows the service migration from one host (e.g., cloud node) to another physical host (e.g., fog node) [38]. Based on QoE, network conditions, and user characteristics, the ABR service running on a virtualized service at the cloud can be migrated to a given fog node. In this context, migration decisions are directly related to the number of users, user experience, and the heterogeneity found across fog nodes. Besides that, localization features should also be considered, including changes in user locations (i.e., “handover”). Since the mobility patterns are not known *a priori*, the migration timing depends on the inference of user mobility and the video pattern demands. Therefore, as soon as the user moves away from the fog node, the video service must be migrated to a new node to maintain QoE.

Service migration can be divided into: non-live (“cold”) migration, where the virtualization service states are lost, impacting in service availability. On the other hand, live (“hot”) migration maintains the operation and associated states, and the service is provided uninterruptedly. In this article, we are interested in live service migration. Live service migration improves the separation of tenants and the operational management procedures since it is possible to simply migrate the entire OS and the running apps as a single unit [38]. To perform live migration, the virtualization service state (i.e., memory contents and file systems) must be collected, and then the virtualization service is suspended at the cloud. Such state is transferred, and the virtualization service is resumed at the fog [39]. After the complete service migration, the client starts to receive the video from the fog node. There are several benefits of live service migration, such as, energy management, where the energy consumption of servers is optimized for resource utilization; load balancing, where virtualization services are migrated from heavy loaded servers to light loaded ones; and fault-tolerance, improving availability, reliability, maintainability, and performability of physical servers by migrating virtualization service in order to avoid failures [39,40].

Fog computing resources can be used to provide video dissemination with improved QoE support. Since such resources are distributed along multi-tiers as virtualization services on fog nodes, it is necessary an architecture that enables the provisioning of a virtualization service on a given tier. In addition, the networking infrastructure can require modifications in order to adapt to changes in network flows due to service migrations. In this context, the use of SDN features enables fast adaptations on network paths and the conformation of access control lists (ACLs). Finally, a SDN API can support applications for the network monitoring, the discovering of possible congestions, and the mitigation of network issues in a fog scenario.

## 4. Video Optimization Service Migration Evaluation

In this section, we describe the experimental scenario, methodology, and metrics used to evaluate the impact of ABR streaming deployed in different levels of the network edge. Next, we analyze the impact of live VM migration between different ties of an ABR services on the video quality level.

### 4.1. Scenario Description

**Figure 3:**
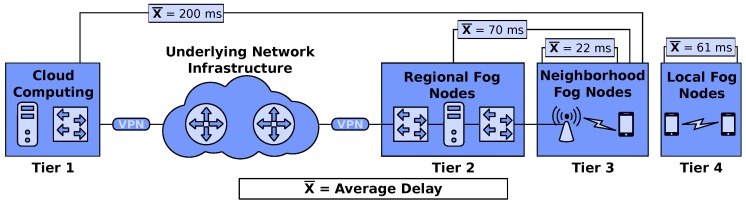
Experimental Scenario.

Figure 3 depicts our experimental scenario composed smartphones, an AP, an ISP, and the cloud to study the performance of ABR streaming unit deployed in fog nodes at different levels of the proposed architecture. We also analyze the impact of service migration between fog nodes at different tiers. Specifically, our experimental scenario considers a Galaxy S7 acting as a fog node in Tier 4, an Icarus Wi-Fi Node [41] as AP in Tier 3, a mini PC operating as a fog nodes in Tier 2, and as a private cloud in Tier 1. We considered a private cloud since we need a more controllable scenario to analyze the results without any external and uncontrollable factors. It is important to mention that the proposed architecture can be used with both private and public cloud infrastructures.

The experimental scenario has the switch Pica8 [42] to connect the devices, considering the learning switch behavior, i.e., it does not have any specific forwarding technic, and thus it operates as a traditional routing. We make use of the floodlight [43] deployed in the cloud to control the switch, and also the service migration. The southbound API must enable the communication between the controller and switch. A Moto Z smartphone acts as the client to download the video and show the content to the user. For the client application at the smartphone, we consider the open-source Google video player, called of Exoplayer [44]. It is a customized video player for Android that implements functions, such as MPEG-DASH, which is not provided by the standard Android video player, i.e., MediaPlayer. It is important to highlight that the proposed architecture is compatible with any video player that provides ABR scheme.

To understand the properties exposed in our experimental scenario, we collected typical delay via CoLisEU [5]. Specifically, the delay between the client and Tier 1 is 200 ms, between the client and Tier 2 is 70 ms, between the client and Tier 3 is 22 ms, and between the client and Tier 2 is 61 ms. In such scenario, we consider a client, i.e., Moto Z, requesting and downloading on-demand video streaming from the stream unit deployed in a given tier of the proposed architecture. The streaming unit is a virtualized service to distribute video content for each client request. We considered the Kernel-based Virtual Machines (KVM) [45] as the virtualization technology, where the KVM is relatively lightweight VM and enable live migration.

The streaming unit provides an ABR service to deliver adapted videos, where the adaptation policy is rate-based, and an AdaptiveEvaluator library available in the Exoplayer estimates the bandwidth. We consider the default buffering parameters provided by Exoplayer, where it always buffers between 15.000 e 30.000 milliseconds (ms). The Exoplayer only starts the video after buffering at least 2.500 ms, which is also the buffering time need to play the video again when the user pauses the video. As soon as the buffer does not have content to render (i.e., stalling event), it has to re-buffer 5.000 ms before to play the video again. In our experimental scenario, the VM can be migrated between tiers by the controller.

We considered the Big Buck Bunny, Sunflower version video downloaded from the video library [46]. Precisely, we used a 4k video with the duration of 635 s, configured with 60 frames per seconds, and encoded into eleven common used resolution and bitrate configurations, as shown in Table 1. In the performed evaluations, the client downloaded the video 30 times from each tier, and the results are provided with a confidence interval of 95%.

We applied well-known objective QoE metrics, namely playback start time and a number of Bitrate Switch [26]. These metrics have a significant influence on user experience, where high values could result in the viewer abandoning the video service completely. Furthermore, we evaluated the QoS in regarding of Round Trip Time (RTT):

- Bitrate and Bitrate Switch Events: the client player tends to start the video by requesting a lower bitrate from the streaming unit, and gradually keeps increasing. The bitrate could later be reduced as soon as the rate of the playback exceeds the buffering rate, due to degraded network conditions. By lowering the bitrate of the video streaming, the client player minimizes the interruptions during the video playback. On the other hand, as soon as the network conditions improve, the video bitrate can be increased. However, frequent switching in bit rates can degrade the QoE. In this way, the initial bitrate, number of bitrate switching events, average bitrate, and final bitrate affect the user experience on consuming video services.
- Playback Start Time: it is the time duration before a video starts to playout, which typically includes the time taken to download the HTML page (or manifest file), load the video player plug-in, and to playback the initial part of the video.
- RTT: time is taken for a packet to be sent plus the time is taken for an acknowledgment that such packet is received.

### 4.2. Impact of ABR Schemes Deployed in Different Tiers of the Proposed Architecture

**Figure 4:**
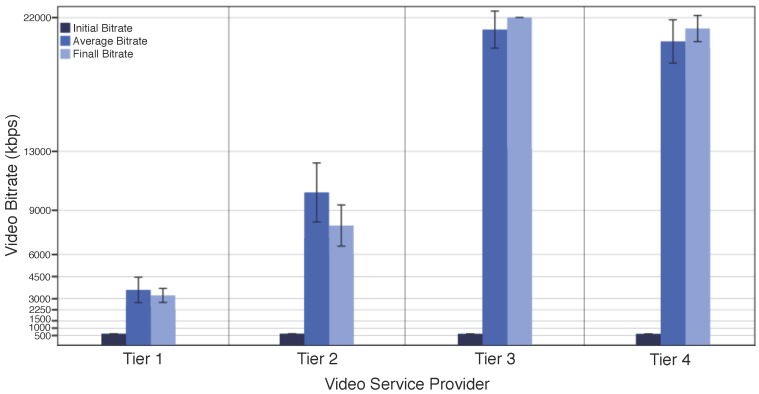
Impact of ABR Schemes Deployed at Different Tiers of the Proposed Architecture.

Figure 4 shows the bitrate received by the client downloading the video deployed in different tiers. As expected in adaptive video streaming, the video has the lower bitrate at the beginning, i.e., 500 kbps, and gradually increases it based on the current network conditions regardless from which Tier the client received the video. The video downloaded from the Tier 1 has an average bitrate lower than 4500 kbps, i.e., Full HD video. This means that even with the video adaptation, the client will not receive higher bit rates than Full HD, or even the video configured in 4K. This behavior is because the network conditions does not allow the video client to request higher bitrates. For instance, the network connection between the client and cloud has a significant impact on the QoE, and users still experience network congestions, longer delays, frequent disconnections. Additionally, thousands of users uploading/downloading multimedia content from the cloud quickly outstrip the bandwidth capacity and increase the delay, reducing the QoE.

The client received the video downloaded from the Tier 2 with a bitrate around 9000 kbps, i.e., Quad HD video. This performance is because fog computing reduces delay by providing computing services closer to the user. For instance, Tier 2 is a fog node deployed in the ISP, and the delay between the client and the Tier 2 is 70 ms, while the delay between the client and the Tier 1 is about 200 ms. The client received a 4K video with the maximum available bitrate by downloading it from Tiers 3 and 4. Specifically, the video downloaded from Tier 3 has a small bitrate gain compared to Tier 4. This bit rate performance is because Tier 3 is composed of a specific hardware to run the ABR unit, while Tier 4 is a smartphone that runs others applications.

Based on the performed experiments, it can be seen that downloading adapted videos from the cloud do not increase the initial, final or average video bitrate compared to downloading the video from fog nodes, since the network conditions, e.g., delay, between the client and cloud impact on the performance of ABR schemes. In this way, it is important to migrate video optimization services from the traditional cloud computing to fog nodes located at the network edge, improving the QoE of delivered videos [30]. This service migration is because fog computing reduces delay by providing computing services closer to the user, and the amount of data uploading/downloading to the cloud for processing and storage. Furthermore, fog computing overcomes bandwidth constraints for long-haul communications and also reduces the processing and energy consumption on mobile devices [14]. Therefore, the cloud and fog nodes must work in a collaborative fashion, where the cloud migrates video optimization services to multi-tier fog nodes.

**Figure 5:**
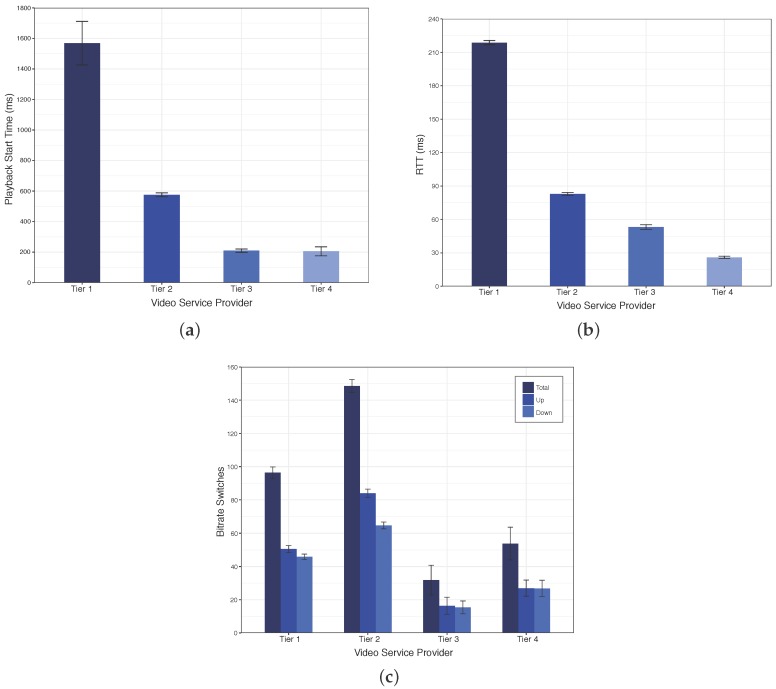
Experimental Results of ABR Schemes Deployed at Different Tiers of the Proposed Architecture. (**a**) Playback Start Time; (**b**) RTT; (**c**) Number of Bitrate Switches.

Figure 5 shows the playback start time, RTT, and a number of bitrate switches for the video downloaded from different tiers. By analyzing the results of Figure 5a, we observe that Tier 1 has large playback start time compared to other tiers, due to Tier 1 has higher typical delay with large variation than other tiers. On the other hand, Tier 2 reduces the playback start time by 60% compared to Tier 1, since the delay between Tier 2 and client is three times less than the between client and Tier 1. Finally, although the RTT for the Tier 3 is larger than Tier 4, its hardware is more robust to deliver video with similar playback start time than Tier 4.

Figure 5b shows the RTT measured in our experiment. The smartphone downloading the video from the Tier 1 has the worst RTT performance. This behavior occurs because the connection between client and Tier 1 mostly impacts the RTT performance, where the typical delay measured in our experimental scenario between smartphone and Tier 1 is about three times higher than between client and Tiers 2/3, as depicted in Figure 3. We also observed that the RTT reduces as soon as the service provider is closer to the network edges since the content is closer to the user.

Figure 5c shows the total number of bitrate switches for the video downloaded from the different tiers, and also the number of times that the bitrate increased and decrease. Tier 1 has less number of bitrate switches compared to Tier 2, since Tier 1 has the higher RTT, and consequently, the client cannot increase the bitrate frequently. On the other hand, we observe that Tier 2 has the higher number of bitrate switches compared to other Tiers. This performance is because the RTT of this tier mainly impacts the bitrate switch events, leading to the client to frequently request to the video provider to increase or to reduce the bitrate. Finally, Tiers 3 and 4 have the lower number of bitrate switches compared to Tiers 1 and 2, since Tiers 3 and 4 quickly achieve the highest bitrate. Furthermore, the client has a right network condition with Tiers 3 and 4, reducing the probability to decrease or increase the bitrate.

### 4.3. Impact of Service Migration between Different Tiers of the Proposed Architecture

We evaluate the impact of VM migration on the video quality level of two scenarios: (i) *Downstream*: the client starts downloading the video from the Tier 1. Afterwards, the controller performs the live VM migration to the Tier 2, and then to Tier 3. (ii) *Upstream*: the client starts downloading the video from the Tier 3. Next, the controller performs the live VM migration to the Tier 2, and then to Tier 1. Specifically, we create and configure a KVM in each tier, to avoid compatibility problems. This configuration is an important issue, since we perform live VM migration by transferring the VM state (i.e., memory contents and file systems), and then the VM is suspended at the cloud. We implemented a Web service in Python considering the libvirt library to perform the VM migration, which requires the source and destination address. In this way, the controller calls this Web service to start the VM migration.

**Figure 6:**
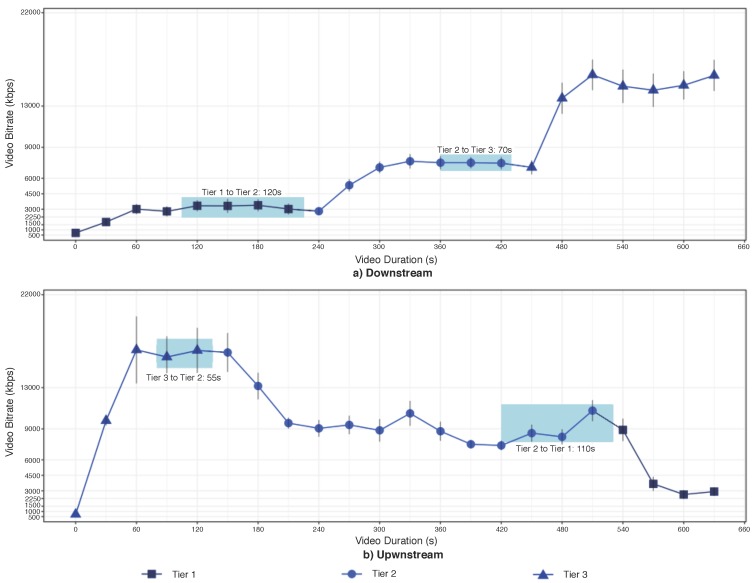
Impact of Live ABR Service Migration Between Tiers of the Proposed Architecture on the Video Bitrate.

Figure 6 depicts the bitrate received by the client downloading a video in a scenario with service migration between different tiers. The controller performs live VM migration, i.e., downstream and upstream, to analyze the impact of VM migration on the video quality level. As expected in ABR video streaming, the video starts with lower bitrate, i.e., 500 kbps, and gradually increases it based on the current network conditions, as shows the results of Figure 6a. However, the client does not receive the video configured in 4K or even with higher bitrates by downloading from the Tier 1. This behavior is because of the network connection between the client and Tier 1, i.e., cloud computing, has the more significant impact on the QoE, since users still experience network congestions, the higher delays, frequent disconnections, which does not allow the client to request/receive higher bitrates.

The controller migrates the streaming unit from Tier 1 to Tier 2, which takes 120 s to migrate the entire OS and the running apps. During the service migration, the client stills downloading the video from Tier 1 with lower bitrate, since we consider live VM migration. After service migration, the VM is suspended at Tier 1, and the client starts to download the video from Tier 2. The bitrate increases by downloading the video from Tier 2 since the network conditions between the client and Tier 2 is better than between the client and Tier 1. However, the client stills not receiving the video configured in 4K. Hence, the controller migrates the stream unit to Tier 3, which takes 70 s. The bitrate increases by downloading the video from Tier 4, and the client receives the video configured in 4k.

By analyzing the upstream results of Figure 6b, we observe that the video starts with lower bitrate, and gradually increases it based on the current network conditions up to the highest bitrate available, i.e., video configured in 4K. To evaluate the impact of upstream on the video quality level, the controller migrates the stream unit from Tier 3 to Tier 2, which takes 55 s. After the VM migration, the bit rate linearly reduces up to 9000 kbps, i.e., Quad HD video, by downloading the video from Tier 2, due to the delay has increased to 70 ms, and thus the client requested lower bitrates to adapt the video transmission based on the network conditions. Afterwards, the controller migrates the stream unit from Tier 2 to Tier 1, which takes 110 s. After the service migration to Tier 1, the bit rate reduces to 3000 kbps, i.e., Full HD video, by downloading the video from Tier 1.

**Figure 7:**
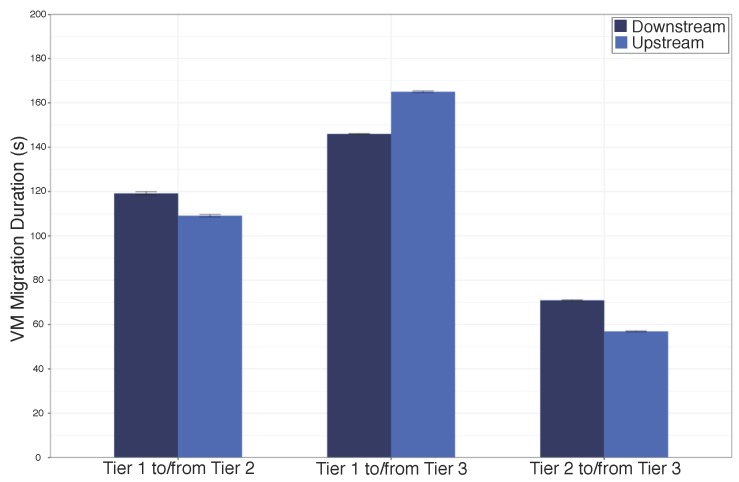
Time Need for Live VM Migration Between Tiers of the Proposed Architecture.

Figure 7 shows the time needed for service migration for both downstream and upstream between different tiers. The time needed to migrate the streaming unit between Tier 1 and 3 is higher compared to the others tiers, since the network connection between the Tier 1 and 3 largely impact on the service migration time. For instance, the delay between the cloud and Tier 3 is about 3 times higher than the between Cloud and Tier 2. On the other hand, the time to migrate the streaming unit between Tiers 2 and 3 is lower, because both tiers are deployed in the network edge with short delay between them. In this way, controller must take into account the time needed to migrate video services in order to improve the user experience on consuming video services.

## 5. Open Issues about Migration of Video Services from the Cloud Computing to Multi-tier Fog Nodes

We identify and discuss a non-exhaustive set of four research challenges coming from the proposed multi-tier fog architecture to provide multimedia dissemination with QoE support, namely orchestration, QoE management, rewarding methods, security, and copyright.

- Orchestration: The Orchestrator might consider information about QoE, QoS, topology, video Content, operator, etc., for service migration, ABR streaming, cache schemes, and other decision-making in such multi-tier fog architecture. The orchestrator must take into account specific algorithms to decide about service migration in the case of poor QoE, where machine learning could improve the orchestrator capabilities. Additionally, orchestrator could combine both ABR and cache schemes in each tier to improve the QoE, beyond that which can be achieved by either ABR and caching running individually. The orchestrator must also deal with users mobility between different locations quite often in the current wireless network scenarios, which hamper the delivery of videos with QoE support. Decision-making schemes based on QoE assessment have a significant advantage to strike an ideal balance between network provisioning and user experience. In this way, a set of different user preferences, interests, devices, video content, and network issues must be modeled and integrated into the orchestrator to optimize and selectively cache or adapt the video content, and also to migrate services. However, it is challenging to integrate QoE assessment, management, and control schemes into the decision-making schemes. Furthermore, the fog architecture could also consider information from different sources, such as social networks, to find users located in the same the area which is sharing similar content.
- Rewarding Methods: It is necessary to design mechanisms for offering incentives to encourage user participation as a fog node, since it consumes computational resources and, consequently, energy. For instance, monetary schemes, coupons, virtual currency, or credit-based incentive mechanisms are the most direct rewarding methods in such case, but factors such as quantization, fairness, and effectiveness must be taken into account. In this context, it lacks a model to describe the best trade-off among rewarding methods, the overall QoE, and the individual contribution of devices for the multimedia system.
- Security: The authorization of the terminal equipment and the mobile subscriber to become a fog node must respect security goals, such as confidentiality, integrity, availability, and accountability. For a fog node to establish a secure communication with a client, some level of pre-provisioning (i.e., bootstrapping) on both nodes is necessary. The minimal number of configuration parameters (e.g., pre-shared keys) for each relay must be configured for each possible peer. This configuration would increase the amount of information the relays may keep for security procedures. On the other hand, cloud-assisted security mechanisms could decrease the required local resources, but they could also increase the execution time for by AAA parties. Consequently, much rests on enabling secure and authenticated multimedia exchanges between fog nodes, which should employ trusted links.
- Copyright: Considering fog nodes, as third-party devices, has implications on copyright issues. Since they can be viewed as parallel and unprotected communication links, as well as they can create threats for the management of digital rights. Therefore, it is also necessary to establish a chain of trust to support this mode of operation in the light of copyright enforcement. Siting between the specified security mechanisms, it is necessary to maintain dependably of the nodes that provide the multimedia content.

## 6. Conclusions

Multimedia traffic on heterogeneous mobile wireless networks is expect to maintain a significant increase in the next years. A high density of users uploading/downloading multimedia content from the cloud can outstrip the bandwidth capacity and incur in the video delivery with poor QoE. Even with C-RANs (and its optimization services), it is necessary technological advances to support this delivery respecting a satisfactory QoE. In this context, fog computing features can provide significant benefits over traditional cloud environments in the context of mobile networking.

In the present article, we described the operational impacts and benefits associated with service migration from the cloud to multi-tier fog nodes in the context of video distribution with QoE support. In this context, we presented a multi-tier fog architecture based on SDN paradigm, which enabled service migration to deliver videos with adequate QoE for mobile users. Besides that, the evaluation of a service migration to minimize the traffic in the core network was introduced. Finally, we introduce trends about service migration between multi-tiers fog nodes, as well as potential research challenges and opportunities.

## Figures and Tables

**Table 1:** Resolution and Bitrate Configurations.

Video Resolution	Video Bitrate
240p	300 kbps
360p	500 kbps
HD 480p	1000 kbps
HD 720p	1500 kbps
HD 720p	2250 kbps
Full HD 1080p	3000 kbps
Full HD 1080p	4500 kbps
Quad HD 1440p	6000 kbps
Quad HD 1440p	9000 kbps
4K UHD 2160p	13,000 kbps
4K UHD 2160p	20,000 kbps
